# Early prediction models for extended-spectrum β-lactamase-producing *Escherichia coli* infection in emergency department: A protocol for systematic review and meta analysis

**DOI:** 10.1097/MD.0000000000025504

**Published:** 2021-04-16

**Authors:** Yiwu Zhou, Shu Zhang

**Affiliations:** Department of Emergency Medicine, Emergency Medical Laboratory, West China Hospital, Sichuan University, Chengdu, Sichuan 610041, China.

**Keywords:** β-lactamase, escherichia coli, extended-spectrum, meta-analysis, prediction models, protocol, systematic review

## Abstract

**Background:**

Resistance in gram-negative bacteria has gained great importance in recent decades and one reason is the rapid increase of extended spectrum β-lactamase (ESBL)-producing bacteria as a growing problem worldwide. The increasing proportion of ESBL-producing Enterobacteriaceae (ESBL-E) infections acquired in the emergency community is a new feature of ESBLs epidemiology. Early recognition of patients with extended-spectrum β-lactamase-producing *Escherichia coli* infection is important in the emergency department. To mitigate the burden on the healthcare system, while also providing the best possible care for patients, early recognition of the infection is needed.

**Methods:**

For the acquisition of required data of eligible prospective/retrospective cohort study or randomized controlled trials (RCTs), we will search for publications from PubMed, Web of science, EMBASE, Cochrane Library, Google scholar. Two independent reviewers will read the full English text of the articles, screened and selected carefully, removing duplication. Then we evaluate the quality and analyses data by Review Manager (V.5.4). Results data will be pooled and meta-analysis will be conducted if there's 2 eligible studies considered.

**Results:**

This systematic review and meta-analysis will evaluate the value of the early prediction models for Extended-spectrum β-lactamase-producing *E coli* infection in emergency department.

**Conclusions:**

This systematic review and meta-analysis will provide clinical evidence for predicting Extended-spectrum β-lactamase-producing *E coli* infection in emergency department, inform our understanding of the value of the predictive model in predicting Extended-spectrum β-lactamase-producing *E coli* infection in emergency department in the early stage. The conclusions drawn from this study may be beneficial to patients, clinicians, and health-related policy makers.

**Study registration number:**

INPLASY202130049.

## 1 Introduction

Antimicrobial resistance is an ecological problem that occurs worldwide and has been associated with increased resistance in both hospital-acquired and community-acquired infections.^[1,2]^ Resistance in gram-negative bacteria has gained great importance in recent decades and one reason is the rapid increase of extended spectrum β-lactamase (ESBL)-producing bacteria as a growing problem worldwide.^[3–5]^ High prevalence rates of ESBL-producing Enterobacteriaceae (ESBL-E) have been reported in *Escherichia coli* and *Klebsiella pneumoniae*.^[6–8]^ Moreover, ESBL presence in Enterobacteriaceae has been associated with high proportions of resistance to non-β-lactam classes of antibiotics as well.^[9,10]^

The increasing proportion of ESBL-E infections acquired in the emergency community is a new feature of ESBLs epidemiology.^[11–13]^ The main infection sites of emergency ESBL-E are urinary tract infections and bloodstream infections.^[14]^ ESBLs are produced by *E coli* and K pneumoniae. Klebsiella has a high incidence in both hospital and community-acquired urinary tract infections which has become the main pathogen of community-acquired bloodstream infections and urinary tract infections.^[11,15]^

The risk factors for ESBL-E infection in the emergency department mainly include advanced age, history of repeated use of antibacterial drugs, invasive procedures, hospital or nursing home residence history, immunosuppression, etc.^[16,17]^ Early recognition of patients with extended-spectrum β-lactamase-producing *E coli* infection is important in the emergency department. To mitigate the burden on the healthcare system, while also providing the best possible care for patients, early recognition the infection is needed. Prediction models that combine several variables or features to estimate the risk of people being infected from extended-spectrum β-lactamase-producing *E coli* could assist medical staff in triaging patients when allocating limited healthcare resources.^[18–21]^ Models based scoring systems have been proposed and published in response to a call to share extended-spectrum β-lactamase-producing *E coli* infection research findings rapidly and openly to inform the public health response and help save lives.^[22–24]^

While up to now, no systematic review or meta-analysis has been performed on models for predicting extended-spectrum β-lactamase-producing *E coli* infection. We aimed to systematically review and critically appraise currently available prediction models for extended-spectrum β-lactamase-producing *E coli* infection.

## 2 Methods

### 2.1 Study registration

This systematic review was recorded in the international platform of registered systematic review and meta-analysis protocols (INPLASY) on March 15, 2021 with the registration number of INPLASY202130049 (doi:10.37766/inplasy2021.3.0049). The protocol will follow the PRISMA protocols 2015 statement which is represented for Preferred Reporting Items for Systematic Review and Meta-Analyses (PRISMA-P).^[25]^

### 2.2 Eligibility criteria for study selection

We will collect all cross-sectional studies that predict or analyzed the patients with Extended-spectrum β-lactamase-producing *E coli* infection in emergency department. The study includes adolescent participants, but children or pregnant women doesn’t meet the inclusion criteria. Animal studies, cadaver studies, comments, case reports, letters, protocols, guidelines, unpublished articles, and review papers will be excluded. No matter where the articles come from, we only review articles in English. Participants who are included in the articles should be diagnosed with Extended-spectrum β-lactamase-producing *E coli* infection. This study will follow the PICOS principle that considering the eligibility criteria adopted in the population of the study, Intervention, Comparison, Result and Study design according to the details showed in the Table 1.

**Table 1.** The PICOS description about study.

PICOS description		
PICOS	Abbreviation	Elements
Patient population	P	Adult
Intervention/Exposure	I	Patients with Extended-spectrum β-lactamase-producing *E coli* infection
Comparison/control	C	Patients without Extended-spectrum β-lactamase-producing *E coli* infection
Outcome	O	Risk of infection due to Extended-spectrum β-lactamase-producing *E coli*
Study design	S	Prospective/retrospective cohort study, RCTs

### 2.3 Search strategy

At first, the collection of bibliographic data will be made in the electronic databases: PubMed, Web of science, EMBASE, Cochrane Library, Google scholar. We use the available publications of the Extended-spectrum β-lactamase-producing *E coli* infection systematic review for a list of keywords. The words are considered: Extended-spectrum β-lactamase-producing, *E coli*, infection, emergency department, Grippe, prognostic, prediction, prediction model, regression, score, artificial intelligence, algorithm, deep learning, machine learning. We make the search terms by combining the words above:

#1 Extended-spectrum β-lactamase-producing, OR ESBL
#2 *Escherichia coli*, OR *E coli*
#3 diagnostic OR imaging OR prognostic OR prognosis OR prediction OR prediction model OR mortality OR regression OR score OR artificial intelligence OR algorithm OR deep learning OR machine learning
#4 emergency department OR ED OR emergency medical service OR emergency room
#5 English NOT animal NOT children NOT child NOT pediatric NOT pediatrics NOT pregnant NOT pregnant woman NOT pregnant women NOT pregnancy NOT gravida NOT gravidity NOT maternal
#1 AND #2 AND #3 AND #4 AND #5

### 2.4 Selection of studies

**Figure 1:**
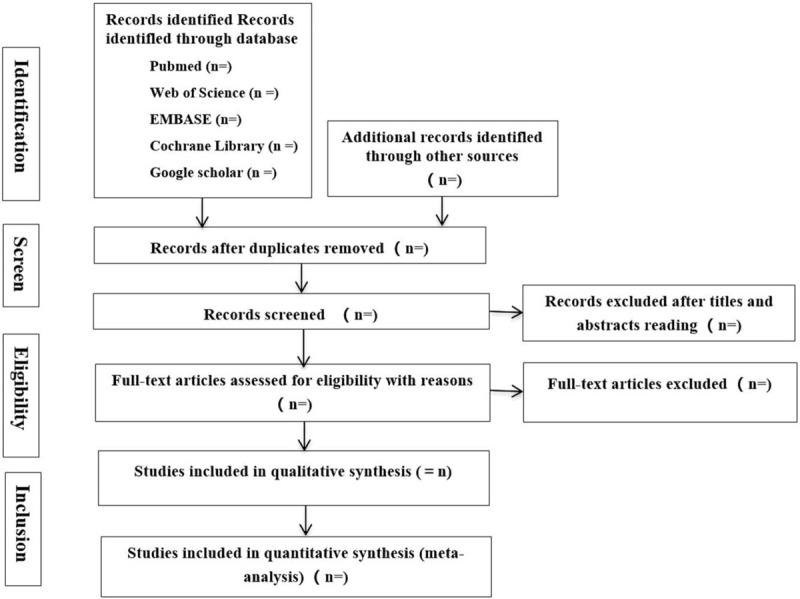
Flow diagram of study selection process.

The preliminary documents are obtained by looking through the titles and abstract, removing the duplications. For the further screening, 2 reviewers will read the full text of the articles which are selected carefully, removing the unsatisfied articles and sending an email to ask author for the full text or the details. Any disagreements will be arbitrated by a third reviewer. The whole process of study selection is presented in the flow chart following the PRISMA principle (Fig. 1).

### 2.5 Data extraction and management

Two of three reviewers will extract the data from eligible studies, putting them into the pre-specified form that we make in advance: author information, study area, study time, study type, study design, setting of study, sample size, participant characteristics, primary and secondary outcomes (needing for mechanical ventilation, needing for ICU care, or dead), AUC (Area Under Curve). Another researcher will solve the divergence between the first two reviewers.

### 2.6 Assessment of risk of bias

The Grading of Recommendations, Assessment, Development and Evaluation (GRADE) assessment tool will be used for conducting an appraisal of the studies’ methodological quality. Every selected study will be evaluated by 2 reviewers independently, a third one as a consulter. The GRADE evaluation system included bias risk; heterogeneity; indirectness; imprecision; publication bias. And each level of evidence is divided into “very ”, “low”, “moderate”, or “high” judgment.

### 2.7 Data synthesis and analysis

For qualified articles, we would like to combine the collected data according to characteristics of eligible trials. In line with the Cochrane guideline, we will express risk ratio with 95% confidence intervals (95%CI) using fixed effect model. Besides the random effect model will be used for continuous outcomes because of clinical heterogeneity. Statistical heterogeneity will be investigated using χ^2^ test and I^2^ statistic (<25%, no heterogeneity; 25%–50%, moderate heterogeneity; and >50%, strong heterogeneity). We will assess possible publication bias using the Egger funnel plot. All data will be performed by using Review Manager (RevMan version 5.4.0) software and *P* value < .5 will be considered statistically significant.

### 2.8 Ethics and dissemination

For the development of this study, approval of ethics and consent is not necessary because it is a systematic review that will use secondary studies. Ethical approval is unnecessary because this is a literature-based study.

## 3 Discussion

To our best knowledge, this is the first systematic review and meta-analysis to investigate prediction models for Extended-spectrum β-lactamase-producing *E coli* infection. Five English databases will be searched to avoid missing any potential eligible studies.

Prognostic models for Extended-spectrum β-lactamase-producing *E coli* infection are available and they all appear to show good to excellent discriminative performance. However, some models are at high risk of bias, mainly because of nonrepresentative selection of control patients, exclusion of patients who had not experienced the event of interest by the end of the study, and model overfitting. Therefore, their performance estimates are likely to be optimistic and misleading. Future studies should address these concerns. Sharing data and expertise for development, validation, and updating of Extended-spectrum β-lactamase-producing *E coli* infection related prediction models is urgently needed.

## Author contributions

**Conceptualization:** Yiwu Zhou.

**Data curation:** Yiwu Zhou.

**Formal analysis:** Yiwu Zhou.

**Funding acquisition:** Yiwu Zhou.

**Investigation:** Yiwu Zhou, Shu Zhang.

**Methodology:** Yiwu Zhou, Shu Zhang.

**Project administration:** Shu Zhang.

**Resources:** Yiwu Zhou.

**Software:** Yiwu Zhou.

**Supervision:** Yiwu Zhou.

**Validation:** Yiwu Zhou.

**Visualization:** Yiwu Zhou.

**Writing – original draft:** Yiwu Zhou.

**Writing – review & editing:** Yiwu Zhou, Shu Zhang.
